# Association of Coagulation Activation with Clinical Complications in Sickle Cell Disease

**DOI:** 10.1371/journal.pone.0029786

**Published:** 2012-01-11

**Authors:** Kenneth I. Ataga, Julia E. Brittain, Payal Desai, Ryan May, Susan Jones, John Delaney, Dell Strayhorn, Alan Hinderliter, Nigel S. Key

**Affiliations:** 1 Division of Hematology/Oncology, University of North Carolina, Chapel Hill, North Carolina, United States of America; 2 Department of Biochemistry and Biophysics, University of North Carolina, Chapel Hill, North Carolina, United States of America; 3 Department of Biostatistics, University of North Carolina, Chapel Hill, North Carolina, United States of America; 4 Division of Cardiology, University of North Carolina, Chapel Hill, North Carolina, United States of America; University of Sao Paulo – USP, Brazil

## Abstract

**Background:**

The contribution of hypercoagulability to the pathophysiology of sickle cell disease (SCD) remains poorly defined. We sought to evaluate the association of markers of coagulation and platelet activation with specific clinical complications and laboratory variables in patients with SCD.

**Design and Methods:**

Plasma markers of coagulation activation (D-dimer and TAT), platelet activation (soluble CD40 ligand), microparticle-associated tissue factor (MPTF) procoagulant activity and other laboratory variables were obtained in a cohort of patients with SCD. Tricuspid regurgitant jet velocity was determined by Doppler echocardiography and the presence/history of clinical complications was ascertained at the time of evaluation, combined with a detailed review of the medical records.

**Results:**

No significant differences in the levels of D-dimer, TAT, soluble CD40 ligand, and MPTF procoagulant activity were observed between patients in the SS/SD/Sβ^0^ thalassemia and SC/Sβ^+^ thalassemia groups. Both TAT and D-dimer were significantly correlated with measures of hemolysis (lactate dehydrogenase, indirect bilirubin and hemoglobin) and soluble vascular cell adhesion molecule-1. In patients in the SS/SD/Sβ^0^ thalassemia group, D-dimer was associated with a history of stroke (p = 0.049), TAT was associated with a history of retinopathy (p = 0.0176), and CD40 ligand was associated with the frequency of pain episodes (p = 0.039). In multivariate analyses, D-dimer was associated with reticulocyte count, lactate dehydrogenase, NT-proBNP and history of stroke; soluble CD40 ligand was associated with WBC count and platelet count; and MPTF procoagulant activity was associated with hemoglobin and history of acute chest syndrome.

**Conclusions:**

This study supports the association of coagulation activation with hemolysis in SCD. The association of D-dimer with a history of stroke suggests that coagulation activation may contribute to the pathophysiology of stroke in clinically severe forms of SCD. More research is needed to evaluate the contribution of coagulation and platelet activation to clinical complications in SCD.

## Introduction

Patients with sickle cell disease (SCD) have an increased risk of stroke, pulmonary hypertension, avascular necrosis of large joints, and pregnancy-related complications, all of which may be due, at least in part, to thrombotic vascular occlusion [1]. They also appear to have a higher risk of pulmonary embolism as well as pregnancy-related venous thromboembolism compared with race-matched controls without SCD [2], [3]. In addition to these thrombotic complications, patients with SCD manifest alterations in platelet function, as well as changes in the procoagulant, anticoagulant and fibrinolytic systems in the direction of a procoagulant phenotype [4]. Furthermore, tissue factor (TF) antigen and TF procoagulant activity are reported to be elevated in the circulation of SCD patients when compared with normal controls [5]–[7]. As a result of these findings, SCD is frequently referred to as a “hypercoagulable state.”

Despite the abundant laboratory evidence of hypercoagulability observed in these patients, it still remains uncertain whether the observed platelet activation, as well as increased thrombin and fibrin generation contributes to the vascular occlusive episodes that characterize SCD or are rather simple epiphenomena.

In the present study, we sought to evaluate the association of markers of coagulation and platelet activation with specific SCD-related clinical complications and laboratory variables in a cohort of patients followed at an adult sickle cell clinic. In addition, we evaluated the relationship between microparticle-associated tissue factor procoagulant activity, plasma markers of coagulation activation and platelet activation.

## Methods

### Patients and Study Design

The study subjects represent a cohort of patients followed at the Adult Sickle Cell Clinic at the University of North Carolina (UNC), Chapel Hill. The data were collected as part of an ongoing study to investigate the pathophysiology of pulmonary hypertension in SCD. Sixty four patients with SCD were included in the analyses that assessed the associations of plasma markers of coagulation and platelet activation with both SCD-related clinical complications and laboratory variables. Patients with SCD were assessed while in the non-crisis, “steady state” (no current acute painful episode or illness and none requiring medical intervention for at least 1 week); had not experienced an episode of acute chest syndrome in the 4 weeks preceding enrollment; and had no clinical evidence of congestive heart failure. None of the patients was on chronic red blood cell transfusion therapy. This study was approved by the Institutional Review Board at UNC, Chapel Hill and all subjects gave written informed consent to participate in accordance with the Declaration of Helsinki.

#### Blood Sample Collection and Preparation

The samples from the study group were obtained during research visits while in the non-crisis, “steady state.” Blood samples were obtained via venipuncture using a 21-gauge needle and drawn into citrate-containing tubes. Plasma samples were prepared in a manner that minimized platelet activation as previously described [8]. The plasma samples were aliquoted and frozen immediately at −80°C for subsequent analysis.

#### Microparticle-Associated Tissue Factor (MPTF) Procoagulant Activity

Platelet-free plasma was obtained from citrated whole blood by centrifugation at 1,500 g for 15 minutes, followed by a clearing spin of 13,000 g for 2 minutes. Microparticles were isolated from platelet-free plasma by centrifugation at 21,000 g for 30 minutes. The pellet was re-suspended in HBSA (20 mM HEPES, pH 7.4, 150 mM NaCl, and 1 mg/ml bovine serum albumin) and re-centrifuged at 20,000 g for 30 minutes, before the final re-suspension in 250 µL HBSA, and MPTF procoagulant activity was measured as previously described using a chromogenic assay [9], [10].

### Plasma Markers of Thrombin Generation, Platelet Activation and Endothelial Activation

Quantification of D-dimer (DiaPharma, Westchester, OH), thrombin-antithrombin (TAT) complexes (Dade Behring, Marburg, Germany), soluble CD40 ligand (R&D systems, Minneapolis, MN) and human soluble vascular cell adhesion molecule-1 (soluble VCAM-1) (R&D systems, Minneapolis, MN) was accomplished using commercially available ELISA kits. Samples were assayed in duplicate and according to manufacturer's instructions. Measurements of N-terminal pro-brain natriuretic peptide (NT-proBNP) and other routine laboratory studies were performed by the McClendon Clinical Laboratory at UNC Hospitals.

### Sickle Cell Disease-Related Clinical Complications

The presence or history of clinical complications in SCD patients was ascertained from a history at the time of evaluation, combined with a detailed review of the medical records. Acute pain episodes (or crises), acute chest syndrome, stroke and other SCD-related complications were defined using standard definitions [11]–[14]. Based on a modification of the definition from both the Cooperative Study of Sickle Cell Disease [11] and the Multicenter Study of Hydroxyurea [12], acute pain episodes referred to episodes that required a visit to a medical facility for acute sickling-related pain for which treatment with a parenterally administered analgesic was needed. Tricuspid regurgitant jet velocity was measured by Doppler echocardiography as previously described [15].

### Statistical Analysis

Wilcoxon rank sum tests were used to compare distributions of continuous variables in two groups. Fisher's exact tests were used to compare categorical variables. Spearman rank correlations were used to identify associations between markers of coagulation activation and specified variables (α = 0.05). Reported p-values are considered ‘nominal’ and are for individual tests, unadjusted for multiple comparisons because of the exploratory nature of this study. Multivariate analyses were performed using multiple regression to assess the associations between clinical and laboratory variables that were found to be associated with markers of coagulation and platelet activation (TAT, D-dimer, soluble CD40 ligand and MPTF procoagulant activity) in univariate analyses (*P*<0·15). Backwards elimination using Akaike's Information Criterion (AIC) was used to select the best model. Statistical analyses were performed using R statistical software (www.r-project.org).

## Results

### Demographic and Laboratory Characteristics

Sixty four patients with SCD (SS: 47; SC: 7; Sβ^0^ thalassemia: 4; Sβ^+^ thalassemia: 5; and SD: 1) were evaluated. Due to the clinical heterogeneity of SCD, the patients were arbitrarily categorized into 2 groups based on presumed disease severity - SS/SD/Sβ^0^ thalassemia and SC/Sβ^+^ thalassemia. The demographic and baseline laboratory characteristics of all the study subjects are shown in Table 1. As expected, patients in the SS/SD/Sβ^0^ thalassemia group had significantly lower hemoglobin values (8.9 g/dL vs. 10.65 g/dL, p<0.0001), but higher platelet counts (412×10^9^/L vs. 320.5×10^9^/L; p = 0.0058), reticulocyte counts (6.6% vs. 3.25%; p = 0.0011), total bilirubin (2.2 mg/dL vs. 0.95 mg/dL; p<0.0001), indirect bilirubin (2.11 mg/dL vs. 0.86 mg/dL; p<0.0001) and lactate dehydrogenase (894 U/L vs. 641 U/L; p = 0.0011) than patients in the SC/Sβ^+^ thalassemia group. No significant differences were observed in the WBC count or in the level of soluble VCAM-1 when patients in both groups were compared.

**Table 1:** Baseline Clinical and Laboratory Characteristics of Study Subjects.

Variable	SS/SD/Sβ^0^ thalassemia (N = 52)	SC/Sβ^+^ thalassemia (N = 12)	p value
Age	37.5 (26.75, 46.25)	49 (30.25, 59.0)	0.068
*Gender: Female	29	7	1.0
*Hydroxyurea therapy (Yes)	34	4	0.054
White Blood Cell (×10^9^/L)	9.1 (7.7, 11.0)	8.65 (4.7, 9.9)	0.28
Hemoglobin (g/dL)	8.9 (8.0, 9.6)	10.65 (9.9, 11.3)	<0.0001
Platelet Count (×10^9^/L)	412 (325, 539)	320.5 (245, 386)	0.0058
Reticulocyte Count (%)	6.6 (4.5, 9.4)	3.25 (1.9, 5.0)	0.0011
Lactate Dehydrogenase (U/L)	894 (700, 1380)	641 (560, 681)	0.0011
Total Bilirubin (mg/dL)	2.2 (1.1, 3.4)	0.95 (0.65, 1.08)	<0.0001
Indirect Biliribin (mg/dL)	2.11 (1.01, 3.21)	0.86 (0.56, 0.98)	<0.0001
Soluble vascular cell adhesion molecule-1 (ng/mL)	1952 (1110, 3188)	1707 (1240, 2544)	0.45
*History of stroke (Yes)	4	1	1.0
*History of avascular necrosis (Yes)	26	5	0.76
*History of leg ulcer (Yes)	12	0	0.10
*Use of hydroxyurea (Yes)	34	4	0.06
*History of retinopathy (Yes)	14	7	0.09
*Pain crisis ≥3 in previous year	15	1	0.15
*History of acute chest syndrome (Yes)	48	7	0.0086
*History of priapism (Yes)	7	0	0.29

Data are presented as medians and interquartile ranges (25^th^ and 75^th^ percentiles).

*The results of a Fishers Exact Test.

### Correlation of Markers of Coagulation and Platelet Activation with Laboratory Variables

**Figure 1:**
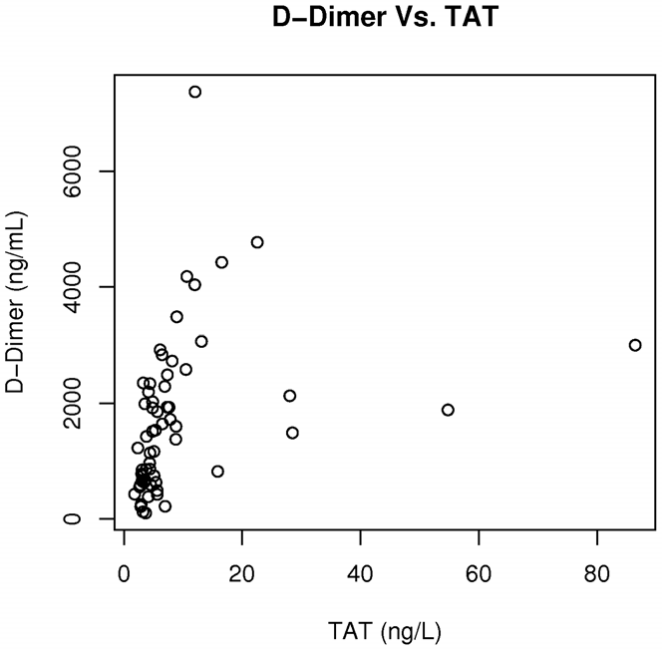
TAT is correlated with D-dimer in sickle cell disease. Plasma TAT is significantly correlated with plasma D-dimer in our study cohort (r = 0.66; p<0.0001).

No significant differences in the levels of D-dimer (1424 ng/mL; interquartile range [IQR], 745–2536 ng/mL vs. 1533.4 ng/mL; IQR, 591–2006 ng/mL, p = 0.47), TAT (5.63 ng/L; IQR, 3.62–8.9 ng/L vs. 4.86 ng/L; IQR, 3.91–6.19 ng/L, p = 0.59), soluble CD40 ligand (0.453 ng/mL; IQR, 0.339–0.60 ng/mL vs. 0.409 ng/mL; IQR, 0.201–0.60 ng/mL, p = 0.24), and MPTF procoagulant activity (0.145 pg/mL, IQR 0.094–0.265 pg/mL, vs. 0.115 pg/mL, IQR 0.056–0.173 pg/mL, p = 0.17) were observed when patients in the SS/SD/Sβ^0^ thalassemia group were compared with patients in the SC/Sβ^+^ thalassemia group. We observed a significant correlation between TAT and D-dimer (r = 0.66; p<0.0001) (Figure 1). However, no correlations were observed between TAT and soluble CD40 ligand (r = 0.016, p = 0.91) or between TAT and MPTF procoagulant activity (r = 0.064, p = 0.63). Similarly, no correlations were observed between D-dimer and soluble CD40 ligand (r = −0.17; p = 0.19) or between D-dimer and MPTF procoagulant activity (r = 0.11, p = 0.40).

**Figure 2:**
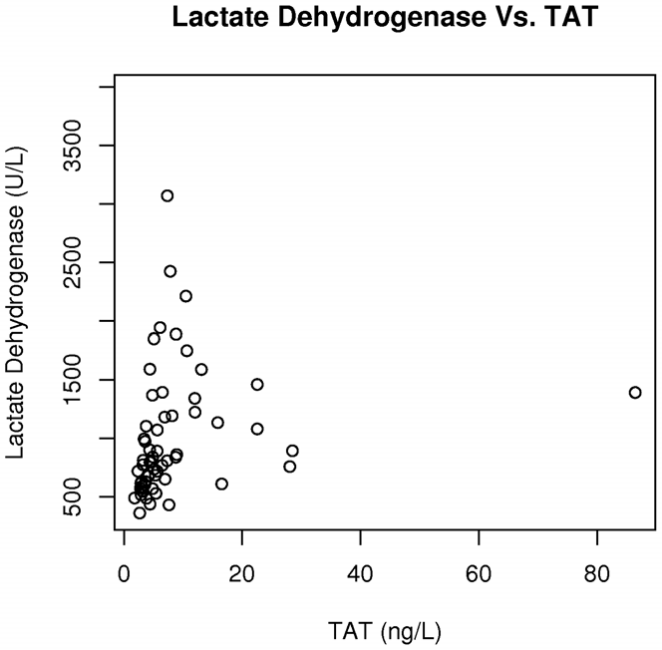
TAT is correlated with lactate dehydrogenase in sickle cell disease. TAT is correlated with lactate dehydrogenase in our study cohort (r = 0.57, p<0.0001).

**Figure 3:**
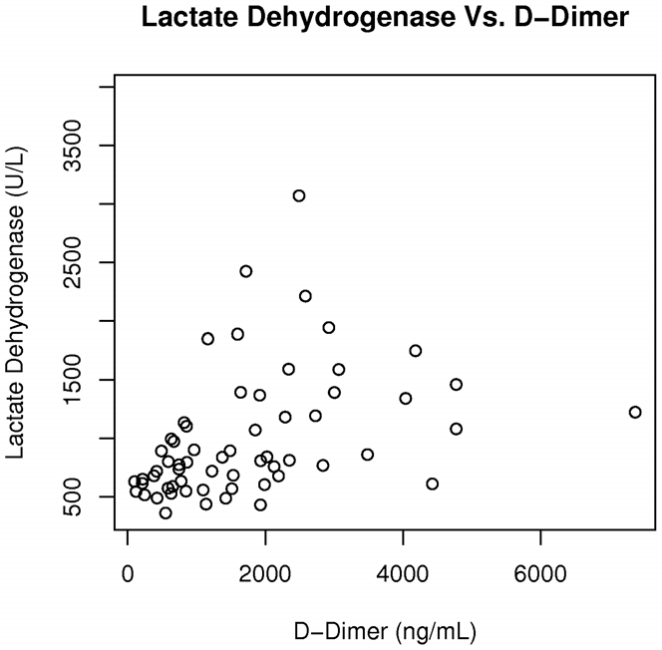
D-dimer is correlated with lactate dehydrogenase in sickle cell disease. D-dimer is correlated with lactate dehydrogenase in our study cohort (r = 0.56; p<0.0001).

**Figure 4:**
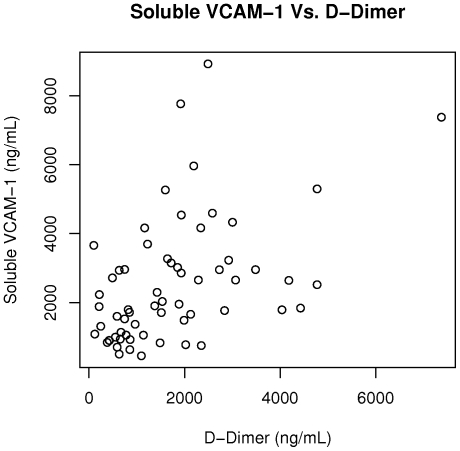
D-dimer is correlated with soluble VCAM-1 in sickle cell disease. D-dimer is correlated with soluble VCAM-1 in our study cohort (r = 0.49; p<0.0001).

The plasma markers of coagulation activation, TAT and D-dimer, were associated with laboratory measures of hemolysis (Table 2). There was a correlation between TAT and lactate dehydrogenase (r = 0.57, p<0.0001) (Figure 2), with borderline correlations between TAT and total bilirubin (r = 0.25; p = 0.054), indirect bilirubin (r = 0.26, p = 0.051), and hemoglobin (r = −0.24; p = 0.071). TAT was also correlated with the absolute monocyte count (r = 0.27; p = 0.035) and NT-proBNP (r = 0.35; p = 0.005). Similarly, D-dimer was correlated with lactate dehydrogenase (r = 0.56; p<0.0001) (Figure 3), indirect bilirubin (r = 0.26; p = 0.048), hemoglobin (r = −0.32; p = 0.012), NT proBNP (r = 0.42; p<0.0001), with borderline correlations with absolute monocyte count (r = 0.23; p = 0.074). Both TAT (r = 0.37; p = 0.004) and D-dimer (r = 0.49; p<0.0001) (Figure 4) were also correlated with soluble VCAM-1.

**Table 2:** Correlation of Markers of Coagulation Activation with Laboratory Variables.

Biomarker	Laboratory Variable	Number of Patients	r value	95% Confidence Interval	p value
TAT	White blood cell count	60	0.038	−0.242–0.31	0.77
	Absolute neutrophil count	60	−0.078	−0.338–0.19	0.55
	Absolute monocyte count	60	0.273	0.018–0.494	0.035
	Hemoglobin	60	−0.235	−0.457–0.005	0.071
	Platelet count	60	−0.154	−0.401–0.096	0.24
	Reticulocyte count	59	0.195	−0.041–0.412	0.14
	Hemoglobin F	57	−0.165	−0.416–0.112	0.22
	Lactate dehydrogenase	60	0.57	0.376–0.717	<0.0001
	Total bilirubin	61	0.248	0.026–0.45	0.054
	Direct bilirubin	58	0.015	−0.251–0.272	0.91
	Indirect bilirubin	58	0.258	0.028–0.456	0.051
	NT-proBNP	61	0.354	0.129–0.541	0.005
D-dimer	White blood count	61	−0.027	−0.287–0.234	0.83
	Absolute neutrophil count	61	−0.112	−0.381–0.16	0.39
	Absolute monocyte count	61	0.231	−0.038–0.486	0.074
	Hemoglobin	61	−0.318	−0.528–0.074	0.013
	Platelet count	61	−0.193	−0.45–0.078	0.14
	Reticulocyte count	60	0.197	−0.055–0.425	0.13
	Hemoglobin F	58	−0.148	−0.4–0.122	0.27
	Lactate dehydrogenase	61	0.559	0.372–0.697	<0.0001
	Total bilirubin	62	0.236	0.015–0.436	0.065
	Direct bilirubin	59	0.113	−0.188–0.387	0.39
	Indirect bilirubin	59	0.258	0.028–0.466	0.048
	NT-proBNP	62	0.417	0.192–0.6	<0.0001

TAT – Thrombin-antithrombin complexes.

NT-proBNP – N-terminal pro-brain natriuretic peptide.

Soluble CD40 ligand was correlated with WBC count (r = 0.30; p = 0.018) and platelet count (r = 0.40; p = 0.0014), but was not correlated with lactate dehydrogenase, total bilirubin, indirect bilirubin or hemoglobin (Table 3). No correlations were observed between MPTF procoagulant activity and measures of hemolysis or other measured laboratory variables.

**Table 3:** Correlation of Microparticle-Associated Tissue factor Procoagulant Activity and Platelet Activation with Laboratory Variables.

Biomarker	Laboratory Variable	Number of Patients	r value	95% Confidence Interval	p value
MPTF	White blood cell count	59	−0.177	−0.443–0.106	0.18
	Absolute neutrophil count	59	−0.144	−0.413–0.146	0.28
	Absolute monocyte count	59	−0.134	−0.404–0.144	0.31
	Hemoglobin	59	0.15	−0.123–0.409	0.26
	Platelet count	59	−0.159	−0.398–0.091	0.23
	Reticulocyte count	58	−0.063	−0.325–0.208	0.64
	Hemoglobin F	56	0.211	−0.047–0.444	0.12
	Lactate dehydrogenase	59	0.011	−0.255–0.271	0.94
	Total bilirubin	60	0.019	−0.248–0.281	0.89
	Direct bilirubin	57	−0.045	−0.308–0.233	0.74
	Indirect bilirubin	57	0.024	−0.258–0.293	0.86
	NT-proBNP	60	−0.1	−0.352–0.166	0.45
CD40 ligand	White blood count	61	0.303	0.065–0.518	0.018
	Absolute neutrophil count	61	0.215	−0.031–0.448	0.096
	Absolute monocyte count	61	0.079	−0.187–0. 339	0.55
	Hemoglobin	61	−0.096	−0.372–0.182	0.46
	Platelet count	61	0.4	0.168–0.595	0.0014
	Reticulocyte count	60	0.098	−0.17–0.359	0.46
	Hemoglobin F	58	0.023	−0.252–0.299	0.86
	Lactate dehydrogenase	61	−0.08	−0.355–0.197	0.54
	Total bilirubin	62	−0.048	−0.287–0.194	0.71
	Direct bilirubin	59	−0.104	−0.328–0.123	0.43
	Indirect bilirubin	59	−0.015	−0.261–0.238	0.91
	NT-proBNP	62	0.104	−0.172–0.359	0.42

MPTF PCA – Microparticle-associated tissue factor procoagulant activity.

NT-proBNP – N-terminal pro-brain natriuretic peptide.

When the analyses were limited to only patients in the SS/SD/Sβ^0^ thalassemia group, there was a significant correlation between TAT and lactate dehydrogenase (r = 0.62, p<0.0001), indirect bilirubin (r = 0.296, p = 0.044), and NT-proBNP (r = 0.32, p = 0.026), with borderline correlation with total bilirubin (r = 0.28, p = 0.053) and absolute monocyte count (r = 0.25, p = 0.089). Similarly, we observed significant correlations between D-dimer and lactate dehydrogenase (r = 0.60, p<0.0001), hemoglobin (r = −0.29, p = 0.039), fetal hemoglobin (r = −0.31, p = 0.035), and NT-proBNP (r = 0.49, p<0.0001), with borderline correlations with platelet count (r = −0.28, p = 0.053), total bilirubin (r = 0.24, p = 0.093) and indirect bilirubin (r = 0.26, p = 0.07). Soluble CD40 ligand was correlated with platelet count (r = 0.41, p = 0.003), but not correlated with lactate dehydrogenase, total bilirubin, indirect bilirubin or hemoglobin. MPTF procoagulant activity was correlated with hemoglobin (r = 0.31, p = 0.034), but not correlated with measures of hemolysis or other measured laboratory variables.

#### Association of Measures of Coagulation and Platelet Activation with Clinical Complications

The level of TAT was significantly higher in patients with a history of retinopathy compared to those without this complication (6.05 ng/L; IQR, 4.91, 10.98 ng/L vs. 4.52 ng/L; IQR, 3.22, 7.33 ng/L, p = 0.023) (Table 4). In addition, the level of TAT was lower in patients on hydroxyurea therapy compared to those not on such therapy (4.39 ng/L; IQR, 3.25, 8.77 ng/L vs. 6.5 ng/L; IQR, 5.08, 8.76 ng/L, p = 0.044). D-dimer appeared to be associated with a history of stroke (3003.1 ng/mL [FEU]; IQR, 1513, 3067 ng/mL [FEU] vs. 1399.5 ng/mL [FEU]; 648.8, 2217 ng/mL [FEU], p = 0.062), although the difference was not statistically significant. When those patients with a measurable tricuspid regurgitant jet velocity were evaluated (N = 43), there was no correlation between D-dimer and tricuspid regurgitant jet velocity (r = 0.25, p = 0.10). There was an association between MPTF procoagulant activity and a history of acute chest syndrome (0.1445 pg/mL; IQR, 0.11, 0.268 pg/mL vs. 0.07 pg/mL; IQR, 0, 0.149 pg/mL, p = 0.045) (Table 5). Finally, soluble CD40 ligand appeared to be associated with the frequency of pain episodes (<3 episodes vs. ≥3 episodes in the past year: 0.406 ng/mL; IQR, 0.428, 0.677 ng/mL vs. 0.498 ng/mL; IQR, 0.292, 0.579 ng/mL, p = 0.058), although the difference was not statistically significant.

**Table 4:** Association of Markers of Coagulation Activation with Clinical Variables.

Biomarker	Clinical Variable	N	Yes (Median, IQR)	N	No (Median, IQR)	p value
TAT (ng/L)	History of stroke	5	13.1 (4.83, 22.55)	55	5.09 (3.62, 7.73)	0.25
	Avascular necrosis	31	5.33 (3.66, 8.30)	30	5.35 (3.70, 9.90)	0.66
	History of leg ulcer	12	5.52 (3.73, 8.73)	48	4.95 (3.36, 6.42)	0.45
	Use of hydroxyurea	36	4.39 (3.25, 8.77)	25	6.5 (5.08, 8.76)	0.044
	History of retinopathy	20	6.05 (4.91, 10.98)	39	4.52 (3.22, 7.33)	0.023
	Pain crisis ≥3 in previous year	15	5.62 (4.26, 12.31)	40	5.38 (3.73, 7.92)	0.70
	History of acute chest syndrome	53	5.42 (3.75, 8.81)	8	4.84 (3.56, 7.13)	0.47
	History of priapism	6	4.81 (3.69, 9.13)	20	5.25 (4.03, 10.98)	0.84
D-dimer (ng/mL [FEU])	History of stroke	5	3003.1 (1513, 3067)	56	1399.5 (648.8, 2217)	0.062
	Avascular necrosis	31	1487 (760.3, 2023)	31	1883.7 (643.4, 2414)	0.72
	History of leg ulcer	12	1269.6 (716.4, 2023)	49	1513.3 (669.7, 2350)	0.77
	Use of hydroxyurea	37	1095.9 (589.4, 2023)	25	1719.4 (1164, 2490)	0.14
	History of retinopathy	20	1626.4 (827.2, 2878)	40	1300.2 (648.8, 2168)	0.41
	Pain crisis ≥3 in previous year	16	1235.5 (562.1, 2076)	40	1587.7 (768.5, 2408)	0.41
	History of acute chest syndrome	54	1500.2 (752.3, 2347)	8	1331.8 (467.1, 1953)	0.39
	History of priapism	6	688.5 (600.2, 2123)	20	1325.6 (580.6, 2339)	0.79

TAT – Thrombin-antithrombin complexes.

N – Number of patients.

**Table 5:** Association of Microparticle-Associated Tissue factor Procoagulant Activity and Platelet Activation with Clinical Variables.

Biomarker	Clinical Variable	N	Yes (Median, IQR)	N	No (Median, IQR)	p value
MPTF PCA(pg/mL)	History of stroke	4	0.208 (0.17, 0.23)	55	0.14 (0.087, 0.246)	0.46
	Avascular necrosis	30	0.15 (0.117, 0.215)	30	0.135 (0.051, 0.278)	0.54
	History of leg ulcer	12	0.136 (0.072, 0.26)	47	0.143 (0.091, 0.225)	0.89
	Use of hydroxyurea	35	0.144 (0.115, 0.246)	25	0.14 (0.065, 0.215)	0.52
	History of retinopathy	20	0.136 (0.081, 0.174)	38	0.143 (0.089, 0.262)	0.42
	Pain crisis ≥3 in previous year	14	0.144 (0.117, 0.294)	40	0.141 (0.069, 0.219)	0.42
	History of acute chest syndrome	52	0.145 (0.11, 0.268)	8	0.07 (0, 0.149)	0.045
	History of priapism	6	0.218 (0.144, 0.271)	19	0.14 (0.105, 0.225)	0.48
CD40 ligand (ng/mL)	History of stroke	5	0.487 (0.409, 0.543)	56	0.444 (0.309, 0.619)	0.76
	Avascular necrosis	31	0.454 (0.354, 0.627)	31	0.424 (0.308, 0.544)	0.39
	History of leg ulcer	12	0.468 (0.404, 0.552)	49	0.43 (0.302, 0.624)	0.53
	Use of hydroxyurea	37	0.429 (0.308, 0.624)	25	0.447 (0.378, 0.575)	0.92
	History of retinopathy	20	0.398 (0.282, 0.628)	40	0.462 (0.373, 0.597)	0.28
	Pain crisis ≥3 in previous year	16	0.498 (0.428, 0.677)	40	0.406 (0.292, 0.579)	0.058
	History of acute chest syndrome	54	0.453 (0.334, 0.615)	8	0.36 (0.214, 0.474)	0.19
	History of priapism	6	0.43 (0.401, 0.516)	20	0.416 (0.285, 0.667)	0.84

MPTF PCA – Microparticle-associated tissue factor procoagulant activity.

N – Number of patients.

When the analyses were limited to patients in the SS/SD/Sβ^0^ thalassemia group, we observed associations between D-dimer and a history of stroke (3035.2 ng/mL [FEU]; IQR 2559, 3494 vs. 1269.6 ng/mL [FEU]; IQR 688.4, 2347, p = 0.049), TAT and history of retinopathy (7.145 ng/L; IQR 5.225, 12.01vs. 4.465 ng/L; IQR 3.215, 7.332, p = 0.018), and soluble CD40 ligand and frequency of pain episodes (<3 episodes vs. ≥3 episodes in the past year: 0.398 ng/mL; IQR 0.308, 0.568 ng/mL vs. 0.543 ng/mL; IQR 0.434, 0.730 ng/mL, p = 0.039), but the previously observed association between MPTF procoagulant activity and acute chest syndrome was no longer significant (0.148 pg/mL; IQR 0.115, 0.274 pg/mL vs. 0.094 pg/mL; IQR 0.047, 0.155 pg/mL, p = 0.32). There appeared to be a correlation between tricuspid regurgitant jet velocity and D-dimer (r = 0.3, p = 0.067), although this was not statistically significant.

### Multivariate Analyses

Clinical and laboratory variables were selected for use in the multivariate model via a backwards elimination procedure using AIC as the fit criterion. The clinical variables were history of stroke, retinopathy, avascular necrosis, history of leg ulcers, and history of acute chest syndrome. The laboratory variables were lactate dehydrogenase, hemoglobin, reticulocyte count, white blood cell count, absolute neutrophil count, platelet count, total bilirubin, direct bilirubin, and NT-proBNP (Table 6). In this multivariate model, D-Dimer was associated with reticulocyte count (estimate: 92.56, p = 0.04), lactate dehydrogenase (estimate: 1.22, p = 0.0002), NT-proBNP (estimate: 0.44, p<0.0001) and history of stroke (estimate: 984.01, p = 0.051). This means that for continuous variables such as lactate dehydrogenase, we expect an increase in D-dimer by 1.22 units for every 1 unit increase in lactate dehydrogenase. For binary responses such as history of stroke, the estimated value of 984.01 indicates that having a history of stroke is predicted to result in an increase in D-dimer of 984.01 ng/mL [FEU] compared to no history of stroke. Soluble CD40 ligand was associated with WBC count (estimate: 0.04, p = 0.05), and platelet count (estimate: 0.001, p = 0.0016). Finally, MPTF procoagulant activity was associated with hemoglobin (estimate: 0.024, p = 0.023), and history of acute chest syndrome (estimate: 0.103, p = 0.029).

**Table 6:** Multivariate Analysis.

	Covariate	Number of patients	Estimate	p value
TAT	Intercept	52	3.14	0.099
	Lactate dehydrogenase		0.003	0.082
	History of stroke		4.59	0.13
	History of retinopathy		2.94	0.093
D-dimer	Intercept	53	51.2	0.89
	Reticulocyte count		92.56	0.042
	Lactate dehydrogenase		1.22	0.0002
	Indirect bilirubin		−112.8	0.19
	NT-proBNP		0.438	<0.0001
	History of stroke		984.01	0.051
	History of avascular necrosis		−437.39	0.10
	History of leg ulcer		−566.43	0.10
	History of retinopathy		521.84	0.073
CD40 ligand	Intercept	51	0.032	0.79
	White blood cell count		0.044	0.049
	Absolute neutrophil count		−0.044	0.15
	Platelet count		0.001	0.0016
	Indirect bilirubin		−0.031	0.062
MPTF PCA	Intercept	53	−0.14	0.21
	Hemoglobin		0.024	0.023
	NT-proBNP		0.000	0.16
	History of retinopathy		−0.054	0.13
	History of acute chest syndrome		0.103	0.029

TAT – Thrombin-antithrombin complexes.

MPTF PCA – Microparticle-associated tissue factor procoagulant activity.

NT-proBNP – N-terminal pro-brain natriuretic peptide.

## Discussion

Although SCD is characterized by hypercoagulability [1], [4], the contribution of coagulation and platelet activation to disease pathophysiology remains poorly defined. Plasma levels of TAT, prothrombin fragment 1.2 and D-dimer are increased in patients with SCD during the non-crisis, “steady state” and are further increased during acute pain episodes [16]. D-dimer levels are reported to correlate with the frequency of pain episodes measured during the following year [16] as well as the interval for development of pain episodes, suggesting that coagulation activation may contribute to vaso-occlusion in SCD. Platelets are also activated in SCD during the non-crisis, “steady state,” with further activation during acute pain episodes [7], [16]. In addition, platelet activation, marked by activated fibrinogen receptor, has been reported to correlate with echocardiography-derived tricuspid jet regurgitant velocity suggesting an association with pulmonary hypertension in SCD [17].

In our present cross-sectional study, we observed no differences in markers of coagulation and platelet activation when patients in the SS/SD/Sβ^0^ thalassemia group were compared to patients in the SC/Sβ^+^ thalassemia group. Patients with sickle cell anemia (HbSS) have previously been reported to have higher plasma levels of markers of coagulation activation compared to patients with HbSC disease [18]. The reason for the absence of a difference between the groups in our study is uncertain, but may be related to the arbitrary grouping of the various genotypes based on the presumed severity of disease. Furthermore, as patients with more “severe” genotypes are more likely to be treated with hydroxyurea, it is possible that the lack of a difference may be due to a decrease in disease severity following hydroxyurea therapy. This presumption is supported by our finding of an association between TAT and hydroxyurea use on univariate analysis. In addition, hydroxyurea has previously been reported to decrease D-dimer levels in patients with SCD [19], suggesting that this drug may decrease the coagulation activation observed in SCD.

As expected, we observed significant correlations between TAT and D-dimer. This is consistent with previous reports in SCD [16]. Similar to previous publications by our group and others [15], [20], our finding of correlations between both TAT and D-dimer with measures of hemolysis extends and confirms the contribution of hemolysis to coagulation activation in SCD. Heme, an inflammatory mediator and a product of hemolysis, has been shown to induce TF expression on the surface of both macrovascular and microvascular endothelial cells in a concentration-dependent manner [21]. In addition, heme produces an upregulation of the expression of TF mRNA, TF protein, and TF procoagulant activity in endothelial cells in a time-dependent manner, effects that may be mediated, at least in part, by the transcription factor, NFkappaB. Thus, heme-induced endothelial TF expression may provide a pathophysiologic link between hemolysis and the coagulation activation in SCD. Unlike a previous report that suggested an association between hemolysis and platelet activation [17], no correlation was observed between soluble CD40 ligand and measures of hemolysis in our study. Surprisingly, no correlations were observed between the measured plasma markers of coagulation activation (TAT and D-dimer) with either soluble CD40 ligand or MPTF procoagulant activity in our study. As CD40 ligand is known to induce the expression of TF in SCD [7], the reason for the lack of association between the plasma markers of coagulation activation and soluble CD40 ligand is uncertain. Furthermore, although MPTF procoagulant activity has previously been reported to correlate with plasma markers of coagulation activation in SCD [22], a recent report showed that while whole blood TF procoagulant activity was correlated with TAT, D-dimer, as well as markers of hemolysis, inflammation and endothelial activation, no such correlations were observed between MPTF procoagulant activity and any of these markers [23]. As a result, the contribution of MPTF procoagulant activity to coagulation activation in SCD remains uncertain.

The correlation of both TAT and D-dimer with soluble VCAM-1 suggests a relationship between activation of the coagulation system and endothelial activation in SCD. Furthermore, the correlation of TAT (and possibly D-dimer) with the absolute monocyte count suggests that monocytes may play a role in the coagulation activation in SCD. Monocytes are activated [24], abnormally express TF [5] and are a source of TF-positive microparticles [22] in SCD. There is increasing evidence of a crosstalk between coagulation activation and inflammation [25]. SCD is often referred to as a chronic inflammatory disease [26], [27] and circulating endothelial cells in SCD patients are reported to express TF [28]. While the observed association between plasma markers of coagulation activation and measures of inflammation in our study does not prove causality, the inflammatory state in SCD may indeed contribute to hypercoagulability in SCD. Studies in transgenic SCD mice demonstrate that the induction of ischemia-reperfusion injury by exposure of the mild sickle-cell phenotype, NY1DD mice, to a hypoxic environment followed by a return to ambient air resulted in increased TF expression in the pulmonary veins [29].

The association between D-dimer and a history of stroke suggests that coagulation activation may contribute to the pathogenesis of stroke in SCD. Although the pathophysiology of stroke in SCD is complex, large vessel arterial obstruction with superimposed thrombosis is commonly observed in patients with thrombotic stroke [30]. Despite the absence of a significant association between coagulation activation and echocardiography-derived tricuspid regurgitant jet velocity in our study, autopsy series show the presence of in situ thrombosis in the pulmonary vasculature of patients with SCD-related pulmonary hypertension [31]. A recent study evaluating the relative role played by cells and plasma in the hypercoagulability observed in β thalassemia patients showed evidence of hypercoagulability based on thromboelastometry evaluation, but thrombin generation determined in platelet-poor plasma was not significantly different from healthy individuals [32]. With the known contribution of red blood cells and other cellular elements to hypercoagulability in SCD, this finding suggests that much like in β thalassemia, evaluation of whole blood may be required to accurately demonstrate associations between coagulation activation and clinical complications in SCD. The absence of an association between coagulation activation and echocardiography-derived tricuspid regurgitant jet velocity in our study may also reflect the small number of patients with catheterization-proven pulmonary hypertension in our cohort. Indeed, a recent multicenter study found no evidence of pulmonary hypertension following right heart catheterization in 72 of 96 patients with elevated tricuspid regurgitant jet velocities on echocardiography, suggesting that Doppler echocardiography may overestimate the prevalence of this condition [33].

The association between soluble CD40 ligand and the frequency of pain episodes in the past year in the SS/SD/Sβ^0^ thalassemia group suggests that platelet activation may contribute to the pathogenesis of acute pain episodes in SCD. This finding is consistent with a previous report showing a correlation between platelet procoagulant activity assessed by annexin V binding and the frequency of pain episodes measured during the following year [16]. Platelet-derived CD40 ligand has been shown to induce tissue factor expression, endothelial cell expression of ICAM-1 and B-cell proliferation in SCD [7] and as such may play a role in disease pathophysiology.

Our study has several limitations. The patients were recruited from a specialty clinic at a tertiary care medical center, and may not represent all patients with SCD. The study population is relatively small, while the number of comparisons made is relatively large. We have reported only nominal p-values for each statistical test, with no adjustment made for multiple comparisons, reflecting the exploratory nature of this study. As with all cross-sectional studies, this analysis demonstrates associations, but cannot prove causation.

In summary, our study supports and extends the association of coagulation activation with hemolysis in SCD. TAT was associated with D-dimer, but these markers of coagulation activation were not associated with soluble CD40 ligand or MPTF procoagulant activity. We also show that D-dimer is associated with a history of stroke in patients with clinically severe forms of SCD. More research is needed to evaluate the pathophysiology of coagulation and platelet activation and their contribution to clinical complications in SCD.
